# Allosteric conformational changes of human HBV core protein transform its assembly

**DOI:** 10.1038/s41598-017-01568-9

**Published:** 2017-05-03

**Authors:** Chuang Liu, Guizhen Fan, Zhao Wang, Hong-Song Chen, Chang-Cheng Yin

**Affiliations:** 1 Department of Biophysics, Peking University Health Science Centre, Peking University, Beijing, 100191 China; 2 Institute of Hepatology, Peking University People’s Hospital, Peking University, Beijing, 100044 China

## Abstract

Hepatitis B Virus core protein (HBc) has multiple roles in the viral lifecycle: viral assembly, compartment for reverse transcription, intracellular trafficking, and nuclear functions. HBc displays assembly polymorphism - it can assemble into icosahedral capsid and aberrant non-capsid structures. It has been hypothesized that the assembly polymorphism is due to allosteric conformational changes of HBc dimer, the smallest assembly unit, however, the mechanism governing the polymorphic assembly of the HBc dimer is still elusive. By using the experimental antiviral drug BAY 41-4109, we successfully transformed the HBc assembly from icosahedral capsid to helical tube. Structural analyses of HBc dimers from helical tubes, T = 4 icosahedral capsid, and sheet-like HBc ensemble revealed differences within the inter-dimer interface. Disruption of the HBc inter-dimer interface may likely promote the various assembly forms of HBc. Our work provides new structural insights into the HBV assembly mechanism and strategic guide for anti-HBV drug design.

## Introduction

Biological processes, such as gene replication, transcription and translation, are performed by macromolecular complexes. The correct assembly of macromolecules into a structurally and functionally relevant form is a key step for a biological process to be carried out successfully. Viruses, such as hepatitis B virus (HBV), present as ideal candidates for the study of a range of biological processes, including macromolecular assembly^1^. HBc (or named Cp^2^), is the capsid-forming “core protein” of human HBV, a major pathogen that kills 600,000 people annually^3^. Although excellent vaccines exist, there are no effective cures for extant chronic infections^3, 4^. In addition to capsid formation, HBc plays many essential roles in HBV lifecycle^5–7^, making it an attractive drug target^8–13^.

Wild-type HBc is a 183-residue polypeptide comprising a structured capsid-forming region (residues 1–149) and a basic, nucleic acid-binding domain (residues 150–183)^14–16^. The structured N-terminal region (hereafter referred as HBc) spontaneously self-assembles *in vitro* and *in vivo* to form icosahedral capsid-like particles identical to nucleo-capsids isolated from patient serum^17, 18^. The structure of HBc 1–149 has been characterized within the context of icosahedral capsids, virions, and sheet-like ensembles^14, 17–24^. HBc homodimers comprise two structural domains (Figure S1): helices 3 and 4 from opposing monomers pack together and form a four-helix bundle dimerization interface, whereas helices 1, 2 and 5 pack together around the base of the four-helix bundle to create the hydrophobic core of “contact” domains^17^. Weak inter-dimer interactions between contact domains stabilize HBV capsids^17, 25^.

Multiple thermodynamic and kinetic studies suggest that HBc has a very malleable structure, with this structural plasticity argued to be functionally important^20, 21^. Studies of HBV capsid assembly have inferred the existence of capsid assembly-active (HBc^Act^) and capsid assembly-incompetent (HBc^Inc^) or aberrant (HBc^Abb^) conformations, where HBc^Act^ assemble into the icosahedral capsid and HBc^Inc^/HBc^Abb^ assemble into non-icosahedral forms^10, 11, 20, 22, 23^. As HBc assembly into icosahedral capsid is essential for HBV lifecycle, it is desirable to understand the structural mechanism of HBc assembly. While structural analyses have proposed hydrophobic interactions between HBc dimers may be responsible for HBc assembly^17, 19–23^, insights into the polymorphic nature of HBc assembly remains elusive.

Bay 41-4109, a heteroaryldihydropyrimidine (HAP) derivative, interacts with HBc altering its assembly, making it potentially useful as an antiviral drug^26, 27^. Previously, it has been shown that HAP drugs at stoichiometric levels can facilitate HBc assembly into capsids, while at higher concentrations HAP drugs can induce HBc assembly into aberrant forms, e.g. tube-like structures^10, 24, 27^. It is noteworthy that BAY 41-4109 can exert its effects on assembly polymorphism both before and after assembly either by misdirecting HBc assembly or by transforming the pre-assembled icosahedral particle into aberrant forms^28^. Using BAY 41-4109, we successfully transformed the HBc assembly from an icosahedral capsid ensemble to a helical tube ensemble. Cryo-electron microscopy and helical reconstruction of the tubular HBc ensemble reveals a unique quaternary structure of HBc that differs from the icosahedral capsid and sheet-like ensembles. Intriguingly, the conformational differences of HBc dimers of the tubular ensemble from the icosahedral capsid and the sheet-like ensemble are subtle, with only lateral and rotational helix movement. These subtle conformational changes alter the inter-dimer interactions, leading to a smaller contact area between HBc dimers. This renders the icosahedral capsid thermodynamically unfavourable due to the weakened inter-dimer interactions and drives the HBc assembly into larger ensemble, the helical tube.

## Results

### BAY 41-4109 transforms HBc assembly

HAP drugs can affect HBc assembly in a dose-dependent manner^10, 26–28^. HAPs induce assembly-active states (HBc^Act^) at stoichiometric levels but stabilize non-capsid polymers at higher concentrations^10, 26–28^. Consistent with these findings, we observed that BAY 41-4109 also affects HBc assembly in a dose-dependent manner: (1) in the absence of BAY 41-4109, HBc dimers assemble normally into icosahedral particles with diameters of 31 nm and 28 nm, indicating that free HBc dimers could assemble into both T = 4 and T = 3 icosahedral capsids (Figure S2a). (2) With a BAY41-4109/HBc dimer molar ratio of 1:6, while intact icosahedral particles were still observed, some icosahedral particles with nicks and larger disk-like particles were present, indicating that at this HAP concentration the icosahedral assembly has begun to be less favoured (Figure S2b); (3) With a BAY 41-4109/HBc dimer molar ratio of 1:5, most icosahedral particles disappear and large disk-like particles and sheet-like ensembles are observed (Figure S2c); (4) With a BAY 41-4109/HBc dimer molar ratio of ≥1:3, cone-shaped and tubular structures of various diameters dominate; the length of the tubular structures varies from 0.6 µm to 1.5 µm, and the width of the tubular structures varies from 30 nm to 50 nm (Figure S2d).

### Cryo-electron microscopy of the HBc tubular ensemble reveals the structure of HBc dimers in the tubular ensemble

**Figure 1:**
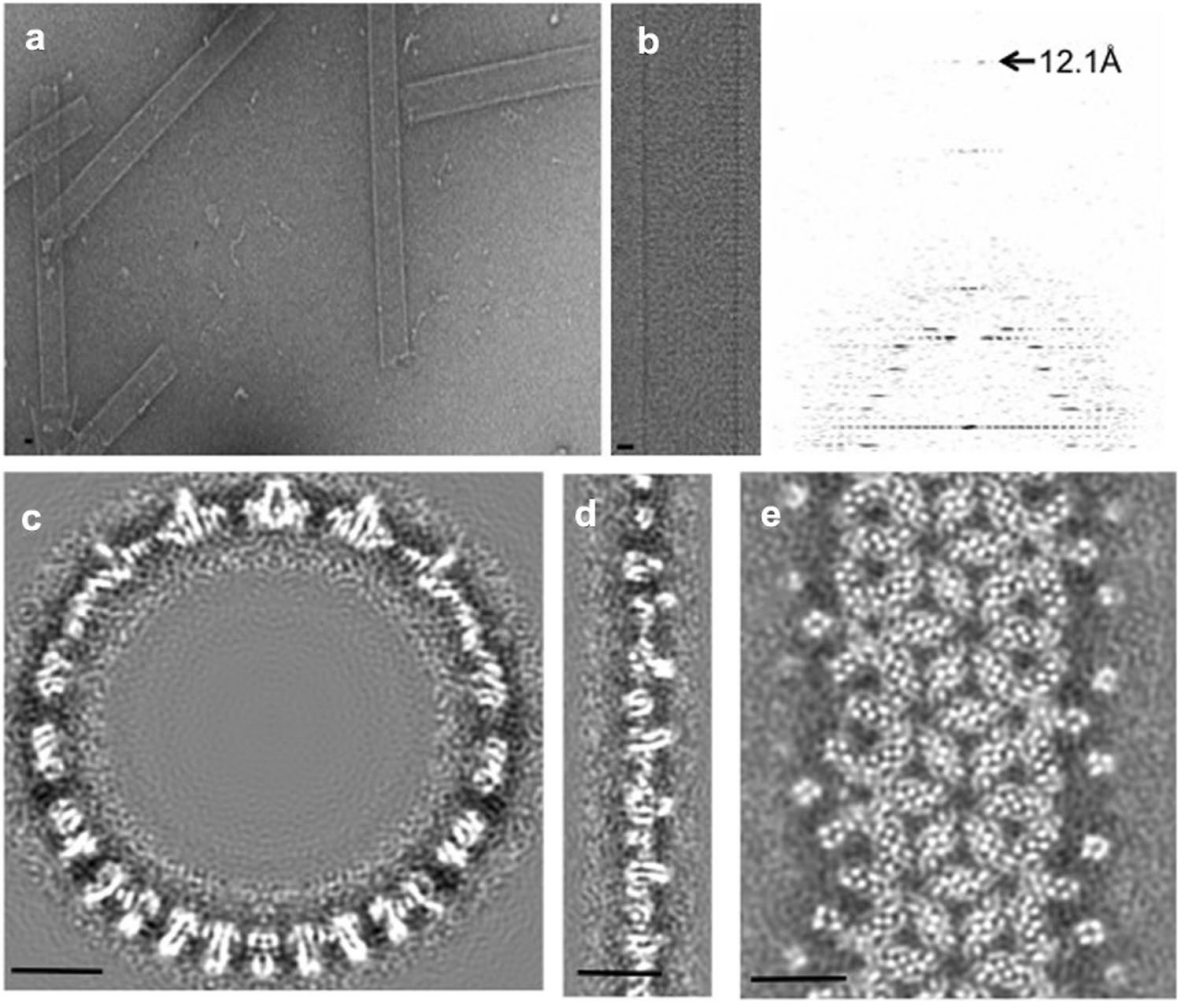
Electron microscopy and 3-D reconstruction of HBc tubular ensembles. (**a**) Electron micrograph of negatively-stained HBc tubular ensembles. (**b**) A representative cryo-EM image of the HBc tubular ensembles and its Fourier transform. The visible diffraction layer lines extend to 12.1 Å. The reconstructed HBc tubular ensemble viewed from its top (**c**), edge (**d**), and side (**e**). In all images, scale bars represent 100 Å.

**Figure 2:**
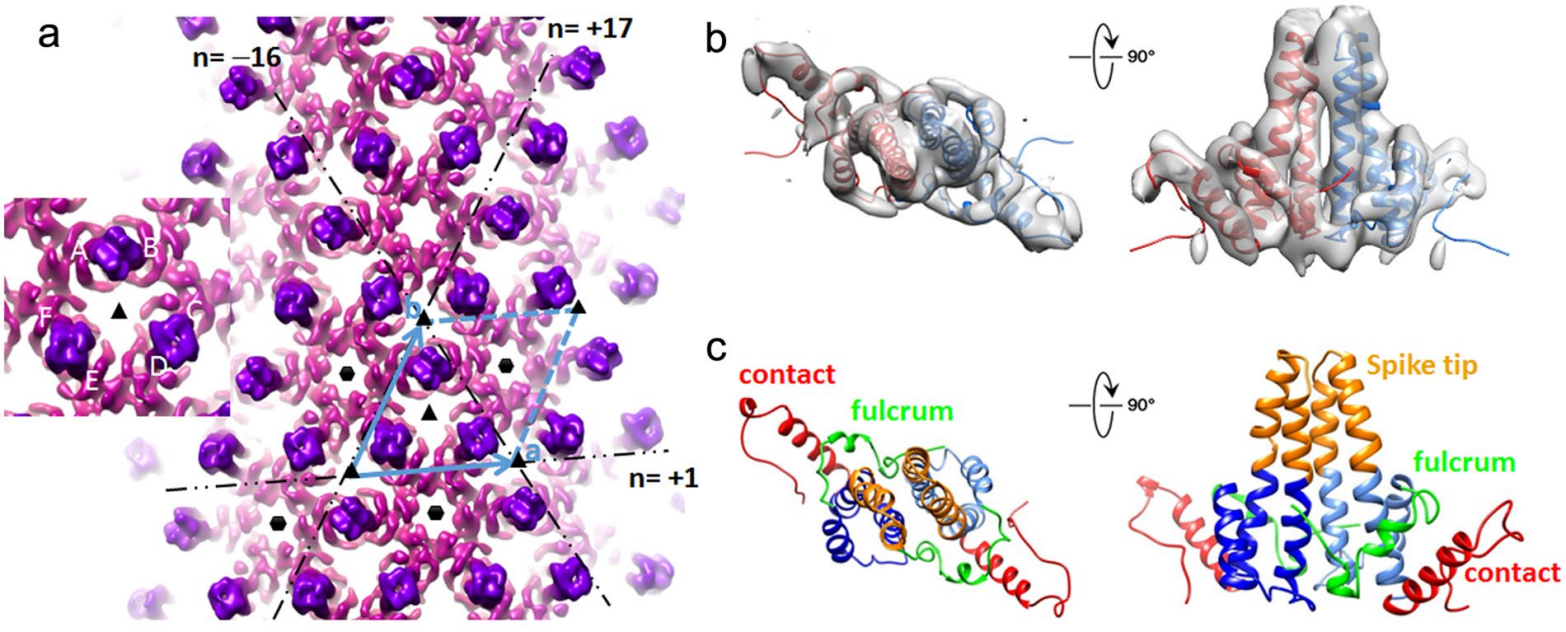
EM density map of HBc tubular ensemble and structural model of HBc dimer in tubular ensemble. (**a**) EM density map of reconstructed HBc tubular ensemble with helical lattice indexes and symmetry elements (quasi 3- and 6-fold axes) marked; the unit cell containing trimer of dimers is framed; the inset is an enlarged version of the trimer of dimer in the unit cell, composed of AB, CD, and EF dimers. (**b**) EM map of segmented AB dimer (transparent grey) with structural model (coloured ribbon) fitted in top (left) and side (right) views. (**c**) Structural model of the AB dimer built by MDFF in top (left) and side (right) views; the structural elements are colour-coded and the structural elements critical for HBc assembly are marked according to the scheme of Packianathan *et al*.^20^.

Previous studies of HBV capsid assembly have proposed the existence of capsid assembly-active (HBc^Act^) and capsid assembly-incompetent (HBc^Inc^) conformations of HBc, in which HBc dimers either assemble into icosahedral capsid or aberrant structures. The HBc in the icosahedral capsid represents the HBc^Act^ form, whereas the HBc in the aberrant structures represents the HBc^Inc^ form. To obtain structural insights into the assembly polymorphism of HBc, it is necessary to compare the structures of HBc^Act^ and HBc^Inc^. However, due to lack of order, the aberrant structures are not amenable for structural analyses. To overcome this difficulty, we targeted the experimental conditions that would favour HBc tubular ensembles (see Methods, Fig. 1a). Cryo-electron microscopy (cryo-EM) of frozen-hydrated HBc tubular ensembles reveal well-ordered helical structures, as manifested by the Fourier transform of the cryo-EM images, which exhibit typical helical diffraction layer lines extending to 12.1 Å resolution (Fig. 1b). A number of helical families with different tube diameters, ranging from 460 to 530 Å, are obtained. We selected 11 of the highest quality images recorded at different defocus values from tubes belonging to helical family (−16, 17) for further structural analyses. Real space three-dimensional (3D) reconstruction was performed by iterative helical real-space reconstruction (IHRSR^29^) and refined with helical constraints and full contrast transfer function (CTF) correction. The final density maps are displayed in Fig. 1c–e. The resolution of the EM density map is estimated as 9.2 Å according to Fourier shell correlation (FSC) 0.5 criterion (Figure S3). At such resolution, secondary structural features, such as α-helices, are clearly identifiable (Figs 1c–e and 2a,b). At sub-nanometer resolution, Cα structural model building is feasible using molecular dynamics flexible fitting (MDFF) approach^30^. We therefore constructed a Cα structural model of the tubular ensemble by MDFF using the crystal structure of the HBc dimer (PDB ID 1QGT) in the T = 4 icosahedral capsid as an initial model.

The HBc tubular ensemble is a single-walled tube with a wall thickness of 47 Å, corresponding to the height of the HBc dimer in the crystal structure of the T = 4 icosahedron; the diameter of the tube is 520 Å; and the dimensions of the plane unit cell are a = 102 Å, b = 104 Å, γ = 62° (Fig. 2a). In our HBc helical ensemble, the unit cell of helical family (−16, 17) is composed of a triangle of three dimers or trimer of dimers (Fig. 2a, inset). The arrangement of trimers of dimers in the lattice displays a network of hexameric rings arrayed with quasi-*p*6 symmetry (Fig. 2a), consistent with the proposal by Stray *et al*. that the HAP drug-induced tube/sheet probably has p6 symmetry^10^.

**Figure 3:**
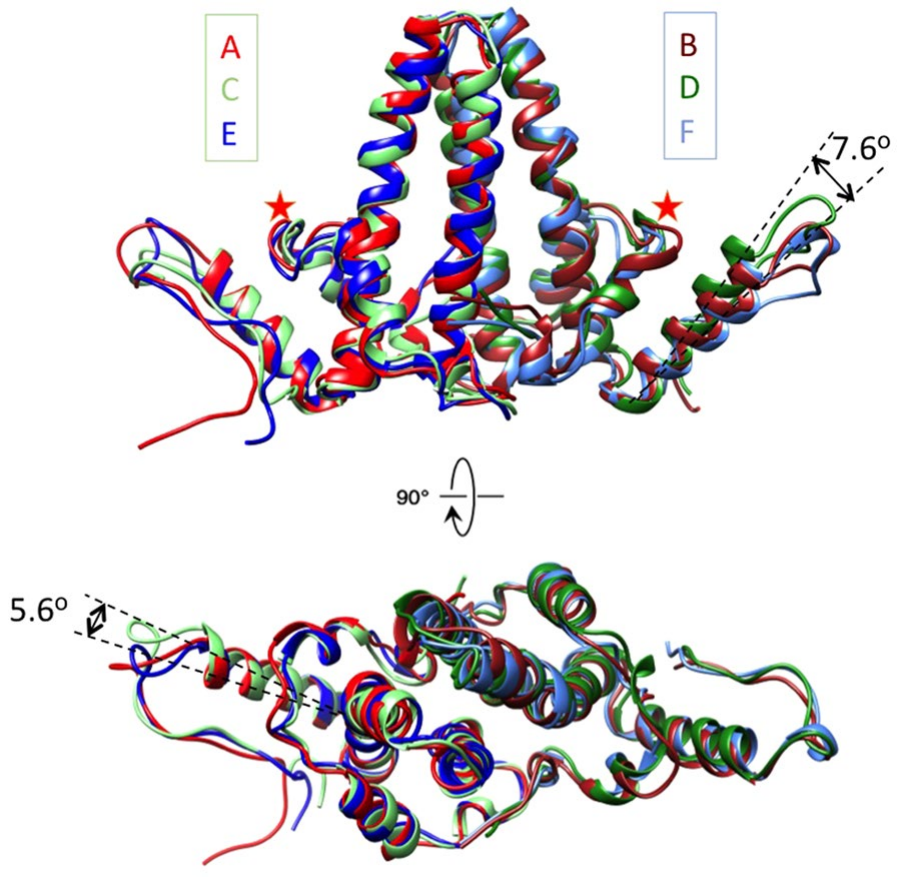
Structural variance of HBc dimers in tubular ensemble. Overlay of structural models of HBc dimers AB (red/brown), CD (light green/green), and EF (blue/cyan) from HBc tubular ensemble in side (upper panel) and top (lower panel) views. Significant conformational differences in helix α5 and helix α1 are indicated by arrows and stars respectively.

The EM densities of three HBc dimers (AB, CD, and EF) in the unit cell of the tube ensemble were segmented and the Cα model for each HBc dimer was built by MDFF (Fig. 2b,c). Although the overall structures of dimers AB, CD, and EF are similar, subtle conformational differences exist between them, particularly in helix α5 (Fig. 3, indicated by arrows) and helix α1 (Fig. 3, indicated by stars); dimers AB and EF have similar conformation but they have significant different conformation from dimer CD in the contact helix α5 (Fig. 3).

### Structural comparison of HBc dimers in different assembly forms

**Figure 4:**
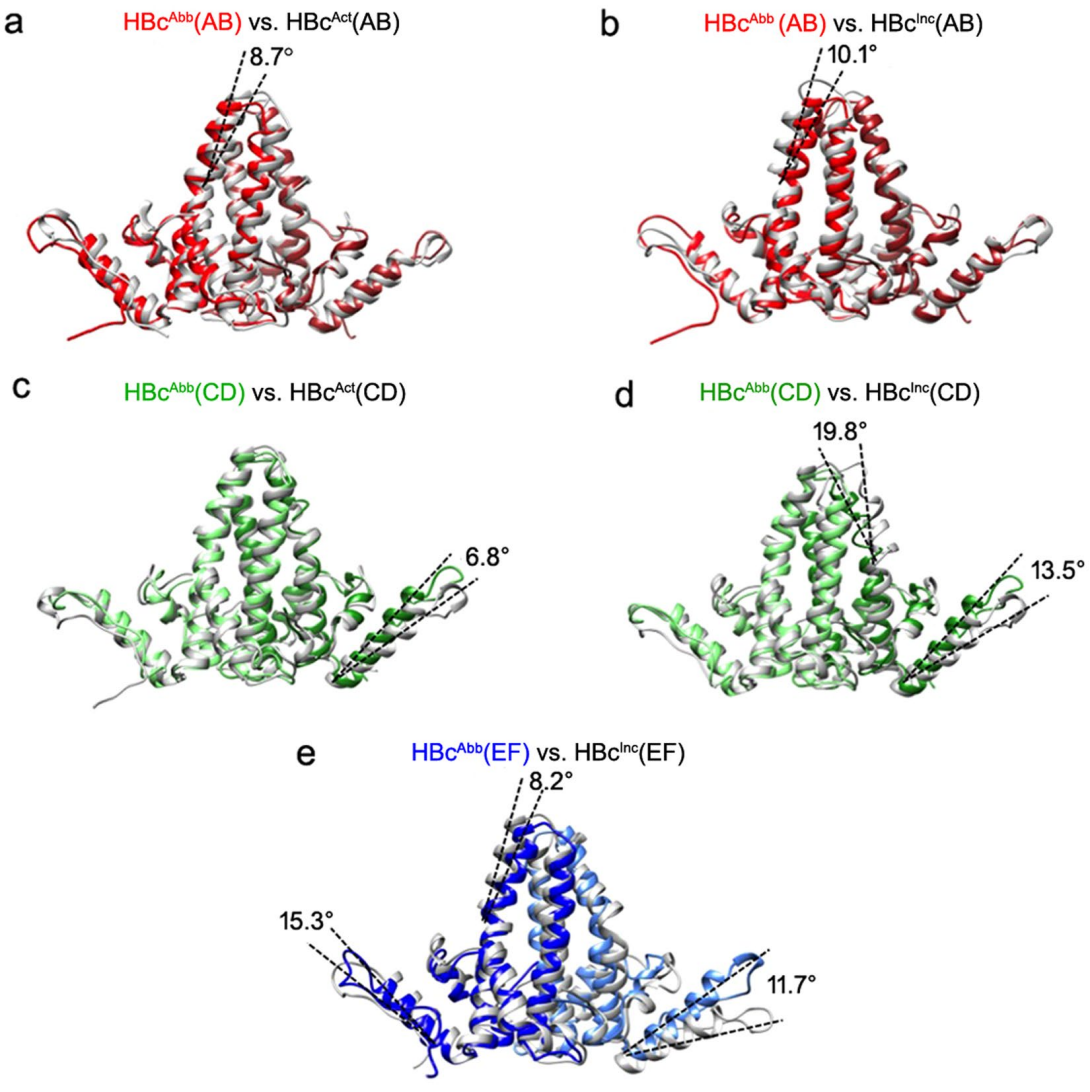
Structural comparison of HBc dimers in tube, capsid and sheet-like ensemble. (**a**,**b**) Overlays of the HBc AB dimer in tube with that in capsid (PDB ID 1QGT) and sheet-like ensemble (PDB ID 3KXS). (**c**,**d**) Overlays of HBc CD dimer in tube with that in capsid (PDB ID 1QGT) and sheet-like ensemble (PDB ID 3KXS). (**e**) Overlay of HBc EF dimer in tube with that in sheet-like ensemble (PDB ID 3KXS). The significant conformational differences are marked with the helix shift angles labelled.

It has been proposed that HBc assembly is allosterically controlled^20^. This implies that different assembly forms, such as icosahedral capsid, tubular ensemble and sheet-like ensemble, may have different conformations. To determine the critical components of the structural polymorphisms we compared the structures of HBc dimers from the tubular ensemble to dimers from the T = 4 icosahedral capsid (PDB ID 1QGT) and sheet-like ensemble (PDB ID 3KXS^20^). To distinguish the different HBc conformations of the three assembly forms, we name the conformation in icosahedral capsid as HBc^Act^, the conformation in sheet-like ensemble as HBc^Inc^, and the conformation in helical tube as HBc^Abb^. Structural overlays of each dimer show considerable differences, indicating their distinct conformations (Figs 4, S4 and S5). In the AB dimer, significant conformational differences are observed for helix α4a when comparing HBc^Abb^ and HBc^Act^ (Fig. 4a) and HBc^Abb^ and HBc^Inc^ (Fig. 4b). Additionally, subtle conformational differences are also observed in the contact helix α5 in the AB dimer. For CD dimer, significant conformational differences are observed in the contact helix α5 between HBc^Abb^ and HBc^Act^ (Fig. 4c), and between HBc^Abb^ and HBc^Inc^ (Fig. 4d); significant conformational difference also exists in helix α4a between HBc^Abb^ and HBc^Inc^ (Fig. 4d). For EF dimer, significant conformational differences exist in the contact helix α5 and helix α4a between HBc^Abb^ and HBc^Inc^ (Fig. 4e). Apart from these significant conformational differences, subtle conformational differences exist in other structural elements throughout the dimers amongst three different assembly forms (Figs 4, S4 and S5).

To determine the critical interfaces between the different assembly forms, we conducted structural analyses of the HBc dimers from capsid, sheet-like and tubular ensembles. We observed that (1) HBc^Act^ dimers within the same ensemble (i.e., apo- or drug-induced capsid) have subtle differences displaying an upward <4° displacement of helix α5 (Figure S6a); (2) HBc^Act^ dimers from different ensembles (i.e., apo- and drug-induced capsid) also have subtle differences, with <4° upward displacement of helix α5 (Figure S6b, Table S1); (3) dimers of the HBc^Inc^ or HBc^Abb^ form within the same ensemble (i.e., tubular or sheet-like ensemble) have significant differences, with a helix α5 upward displacement of >7° in the tubular form and 13° in the sheet-like form (Figs 3 and S6c); (4) the same type of HBc dimers from different assembly forms (i.e., capsid, sheet, tube) also have subtle but major differences, with a helix α5 upward displacement of >6.7° (Figs 4, S4 and S6d) with the largest upward displacement of >15.3° (Fig. 4e). We conclude that subtle but major structural change of helix α5 transforms the HBc assembly from capsid to aberrant forms (sheet or tube).

**Figure 5:**
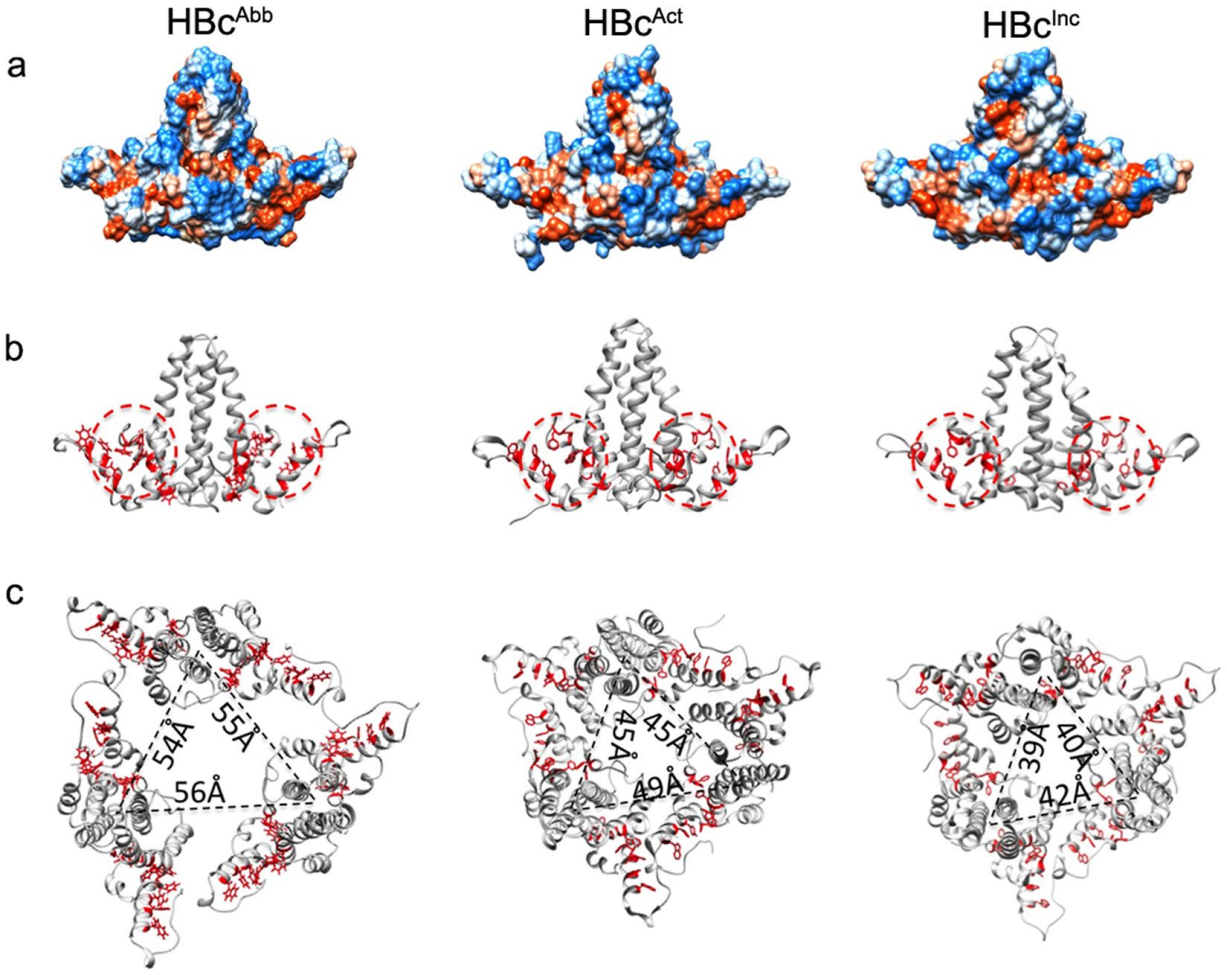
Structural features of HBc dimers in tube, capsid and sheet-like ensemble. (**a**) Structural models of dimers in tube (left), capsid (middle) and sheet-like ensemble (right), displayed as space-filled models with hydrophilic and hydrophobic residues rendered blue and red respectively, or (**b**) depictured as grey ribbon models with hydrophobic residues in helix α5 and helix α1 displayed as red sticks. (**c**) The trimers of dimers in the unit cell/asymmetric unit of HBc tube, capsid and sheet-like ensemble are displayed as grey ribbon models with hydrophobic residues in helix α5 and helix α1 displayed as red sticks. The three inter-dimer distances within the trimer of dimers of tube, capsid and sheet-like ensemble are labelled.

To see how these structural differences affect the assembly of HBc, we then analysed the inter-dimer interactions in these different assembly forms. Compared with the dimers in icosahedral capsid and sheet-like ensemble, the most significant structural change of the dimer in tubular ensemble is an up-ward rotation movement of the contact helix α5 (Figs 4 and S4). Such movement drives helix α5 moving closer to helices α1 and α2a, thus making the individual dimers more compact than the other two forms (Fig. 5a,b); meanwhile, such movement decreases the contact area between neighbouring dimers in the tubular form than in the other two forms, thus making the interaction between neighbouring dimers in tube weaker than the other two forms (Figs 5c and S7).

## Discussion

HBc displays assembly polymorphism but the structural mechanism is elusive. It has been hypothesized that the assembly polymorphism is due to allosteric conformational changes of HBc dimer, the smallest assembly unit^20^. In this work, we analysed the structures of HBc tubular ensemble and the HBc dimers in the context of the tubular ensemble. Structural comparison with other type of high-order assembly forms, including the T = 4 icosahedral capsid and a sheet-like ensemble, revealed that the conformations of HBc dimers in these different HBc assembly forms contain important structural differences that alter the inter-dimer interactions allowing for the formation of alternate assembly forms.

**Figure 6:**
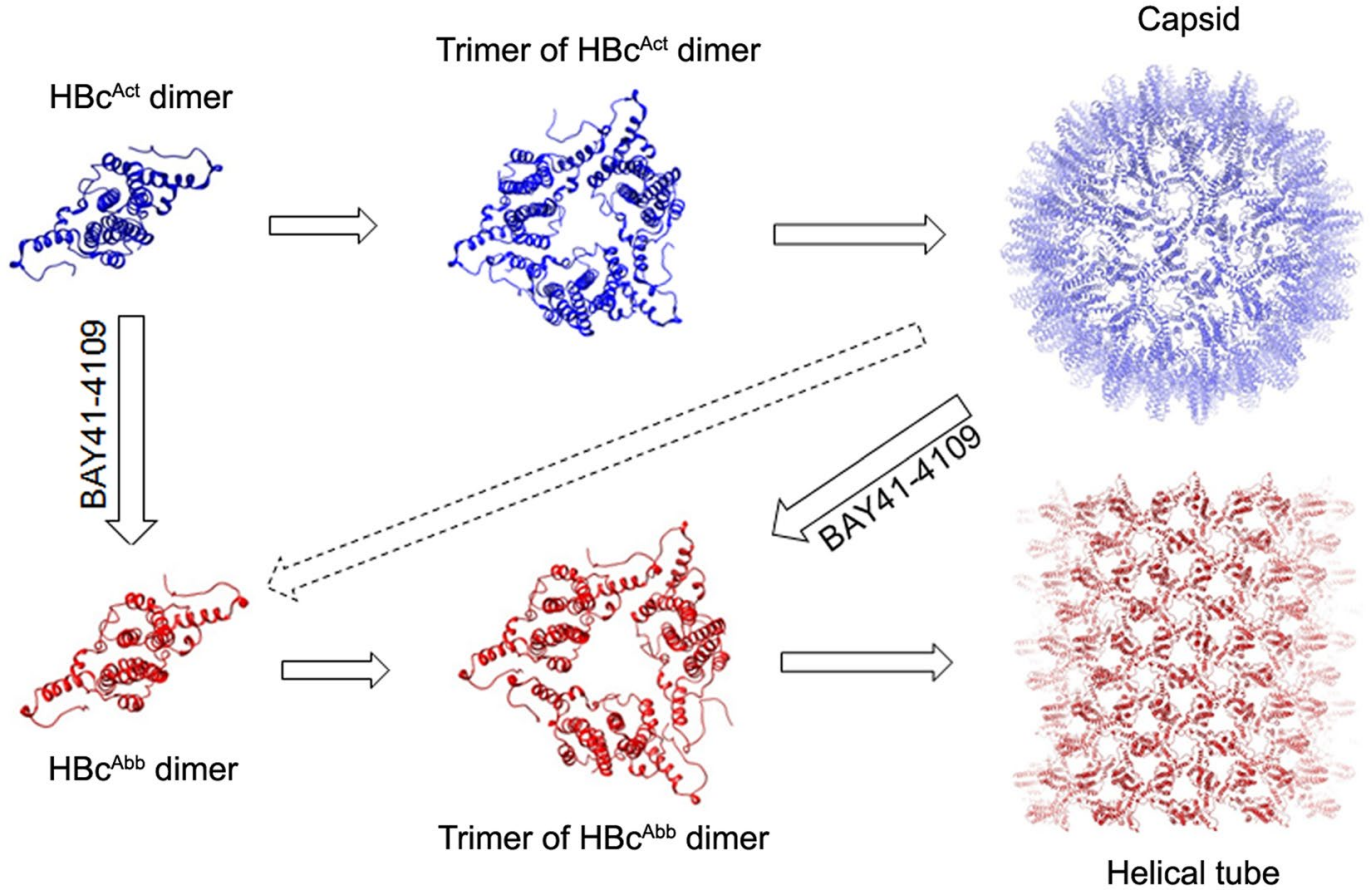
Schematic model of HBc assembly controlled by allosteric conformational changes. In wild-type, the HBc dimer is in the assembly-active (HBc^Act^) conformation and the HBc dimers first assemble into trimer of dimers, in which the interactions between dimers are weak, hence the trimer is thermodynamically unstable therefore the assembly proceeds further to a large, closed ensemble, the capsid (upper panel). BAY41-4109 induces a conformational change of HBc dimer and renders it into an aberrant conformation (HBc^Abb^), the HBc dimers first assemble into trimer of dimers, in which the interactions between dimers are even weaker, hence the trimer is thermodynamically unstable therefore the assembly proceeds further to an even large ensemble, the tube (lower panel). The binding of BAY41-4109 to the preformed capsid can also change the conformation of HBc dimer from HBc^Act^ to HBc^Abb^ and destabilize the capsid, which may render the capsid to dissociate into trimers of dimers or dimers; the trimers of dimers or dimers then re-associate to assemble into a tube.

Based on structural analyses, we propose a mechanism how BAY 41-4109 transforms the HBc assembly from capsid into tube (Fig. 6). In the presence of BAY 41-4109, the drug induces a conformational change within the HBc dimer to promote a structural transfer from HBc^Act^ to HBc^Abb^; by weakening the inter-dimer interactions, and destabilizing the capsid thereby driving the assembly into the helical tube ensemble. Alternatively, BAY 41-4109, which is structurally similar to HAP-1^26^, could bind like HAP-1 at the hydrophobic pocket formed by helices α2, α4b and α5 of HBc and helix α5 of neighbour HBc^27, 31^. Thus BAY 41-4109 induced conformational changes of HBc, particularly the up-ward movement of helix α5, may change the dimer-dimer interaction angle and decrease the curvature of the ensemble, driving the assembly from capsid to tube.

HBc has multiple roles in the HBV viral lifecycle- assembly, compartment for reverse transcription, intracellular trafficking, and nuclear functions^1, 2^, thus, making it an attractive antiviral target. Zlotnick and colleagues propose that the assembly of HBc could be taken as a target for novel antiviral drug design^1, 2^. Our work provides structural insights into HBc assembly, and may serve as a strategic guide for antiviral drug design. HBc assembly into icosahedral capsid is essential for HBV lifecycle; therefore drugs that can misdirect the HBc assembly could be potentially effective in anti-HBV targets. Anti-viral drugs could be designed such that they either enhance the interaction between HBc dimers to trap the ensemble intermediates, or weaken the interactions between HBc dimers, thus misdirecting the assembly into aberrant forms could prove beneficial to HBV treatments.

## Methods

### Drug BAY 41-4109

BAY 41-4109 was obtained from Bayer China Co. The drug was dissolved in dimethyl sulfoxide (DMSO) at 20 mM.

### HBc dimer expression and purification

*E. coli* expressed Human HBc1-149 dimer was purchased from Protgen Ltd. (Beijing). To remove assembly-inactive HBc dimer, an assembly-dissociation step was performed according to Zlotnick *et al*.^32^. Briefly, HBc dimer (1 mg/ml) was first dialysed against reaction buffer (50 mM HEPES, pH 7.5, 2 mM dithiothreitol). Assembly was then initiated by adding NaCl to final concentration of 0.5 M. Assembled capsids were separated from free dimers by size exclusion chromatography (SEC) on a Sephacryl S-300 column equilibrated with reaction buffer plus 0.5 M NaCl and run on a ÄKTA purifier (GE). The capsid fractions were pooled and concentrated to 1 mg/ml by centrifugation in a Millipore concentrator (MWCF 100, Millipore Corp.). Capsids were then dissociated into HBc dimers by adding solid urea to a final concentration of 3.0 M. After incubation for 1.5 h in urea at 4 °C, freshly dissociated dimer was purified by SEC on a Sephacryl S-300 column equilibrated with storage buffer (0.1 M NaHCO_3_, pH 9.6, 2 mM DTT). This material was active in assembly and could be stored at −80° for several months without the loss of assembly activity. Protein was quantified by absorbance by using an ε_280_ of 60,900 M^−1^ cm^−1^ for HBc dimers.

### HBc dimer assembly into tubular ensemble

40 µM human HBc dimer was mixed with 80 µM BAY 41-4109 in pre-assembly buffer (50 mM HEPES, 5 mM DTT, pH 7.5), then the mixture was incubated in water bath at 37 °C for 1 hour to enable Bay 41-4109 binding to HBc dimer. The assembly of HBc into tubular ensemble was initiated by adding NaCl (2 M) to a final concentration of 0.2 M, the system was then incubated in water bath at 37 °C. The HBc dimers assemble into tubular ensembles in 1 hour.

### Cryo-EM specimen preparation and data collection

HBc1-149 dimer tubes (2 µl) were applied to the carbon side of a glow-discharged 400-mesh R1.2/1.3 Quantifoil holy carbon grid (Quantifoil Micro Tools GmbH). The grid was then blotted from the back with a filter paper and plunge-frozen in liquid ethane using a homemade manual gravity plunger. Low dose (15~20 e^−^/Å^2^) images were collected on Kodak SO-163 films with an FEI Tecnai F20 electron microscope (FEI) operated at 200 kV at a nominal magnification of 62,000 and defocus values ranging from 1.5 to 3.0 µm. The best micrographs were digitized using a Nikon super coolscan 9000 ED scanner (Nikon) at a resolution of 4000 dpi, corresponding to 1.06 Å/pixel after calibration.

### Three-dimensional reconstruction

A total of 195 films were recorded. Micrographs in proper defocus range and without apparent specimen drift and charging were used for image processing and 3D reconstruction. Well-ordered tube images were Fourier transformed and indexed for their helical symmetries. Only 11 tubes with the same helical family (−16, 17) were included for further processing and reconstruction. Briefly, the helical tubes were randomly divided into two half groups and 3D maps reconstructed separately. The helical tube images were boxed into small segments of 400 × 400 pixels with an overlap of 95% along the helical axis using SPIDER^33^. The defocus values of images were estimated by EMAN^34^. EM map was calculated and further refined using IHRSR real-space processing package^29^. 4,500 particles were included in the final reconstruction. The B-factor was estimated using FREALIGN and −150 Å^2^ was used to scale amplitudes. The 9.2 Å resolution was calculated by Fourier Shell correlation (FSC) 0.5 criterion. A model vs. map FSC was also calculated, which gives a resolution of 9 Å at FSC 0.5, consistent with the FSC calculated between two independent half-maps. In addition, and more importantly, the feature of the map is consistent with a resolution of 9 Å based on FSC = 0.5, rather than 7 Å based on FSC = 0.143. A hollow cylindrical mask with outer diameter 580 Å and inner diameter 380 Å was used to get rid of most noise while cover all the density of the reconstructed map. To minimize artifacts due to masking, the mask was soft-edged and applied to the reconstructed map before FSC calculation.

### Molecular dynamics flexible fitting

The Cα models for three HBc dimers (AB, CD, EF) in the unit cell of the helical tube were built by molecular dynamics flexible fitting (MDFF^30^) using the HBc dimer of the T = 4 capsid (PDB ID 1QGT) as an initial model. First, the EM density of three HBc dimers (AB, CD, EF) were segmented out, then rigid body docking of the initial model was done using Situs^35^. MDFF simulations were performed using NAMD^30, 36^. In order to optimize bond geometries and avoid clashes in the input model, a multiple time-stepping integration scheme was used by calculating bonded interactions every 1 fs and non-bonded interactions every 2 fs. The temperature was maintained at 300 K using a Langevin thermostat^37^ coupled to all heavy atoms with a damping coefficient of 5 ps. After MDFF, converged structures were obtained and the refined models fit well with the EM density maps. Typical global cross-correlation coefficients obtained improve from 0.7559 to 0.9263 after completion of the flexible fitting.

### Structural analyses

We performed analyses on the structural differences of HBc dimers in capsid, sheet-like and the tubular ensembles. To quantitatively evaluate the structural changes, two structural models were first aligned using ‘*MatchMaker*’ in Chimera based on the minimum RMSD between two models, then the displacement angles of particular helices between two different HBc dimers were calculated. Each angle is computed based on the orientation of the vector lining the helix axis of individual helix using Chimera. The helix axis of individual helix is computed using all coordinates of the C-alpha atoms of the helix (a liner simulation from C-alpha coordinates).

## Electronic supplementary material

Supplementary Info
