# Supplementary material for: Allosteric conformational changes of human HBV core protein transform its assembly
Source: Sci Rep. 2017 May 3;7:1404. doi: 10.1038/s41598-017-01568-9 (PMC5431180; doi:10.1038/s41598-017-01568-9)
Supplement: Supplementary file 1 — Supplementary Info [file 41598_2017_1568_MOESM1_ESM.pdf]

Allosteric conformational changes of human HBV core protein transform its assembly

Chuang Liu[1†], Guizhen Fan[1†], Zhao Wang[1], Hong-Song Chen[2], Chang-Cheng Yin[1*]

[1] Department of Biophysics, Peking University Health Science Centre, Peking University, Beijing 100191, China

[2] Institute of Hepatology, Peking University People's Hospital, Peking University, Beijing 100044, China

[†] These authors contributed equally to this work

[*] To whom correspondence should be addressed. E-mail: ccyin@hsc.pku.edu.cn

Supplementary Material

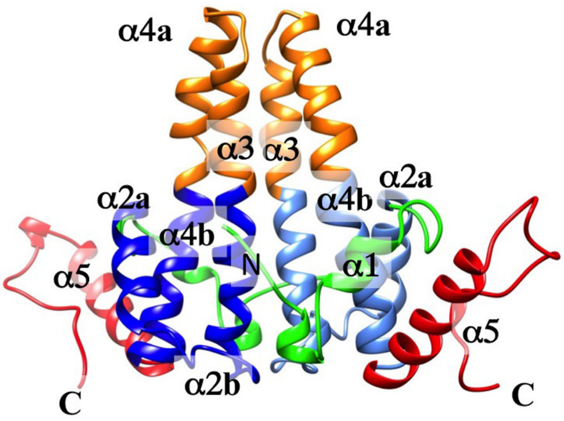

Figure S1. Structural model of HBc dimer with structural elements colour-coded and labelled according to the scheme of Packianathan *et al* [20].

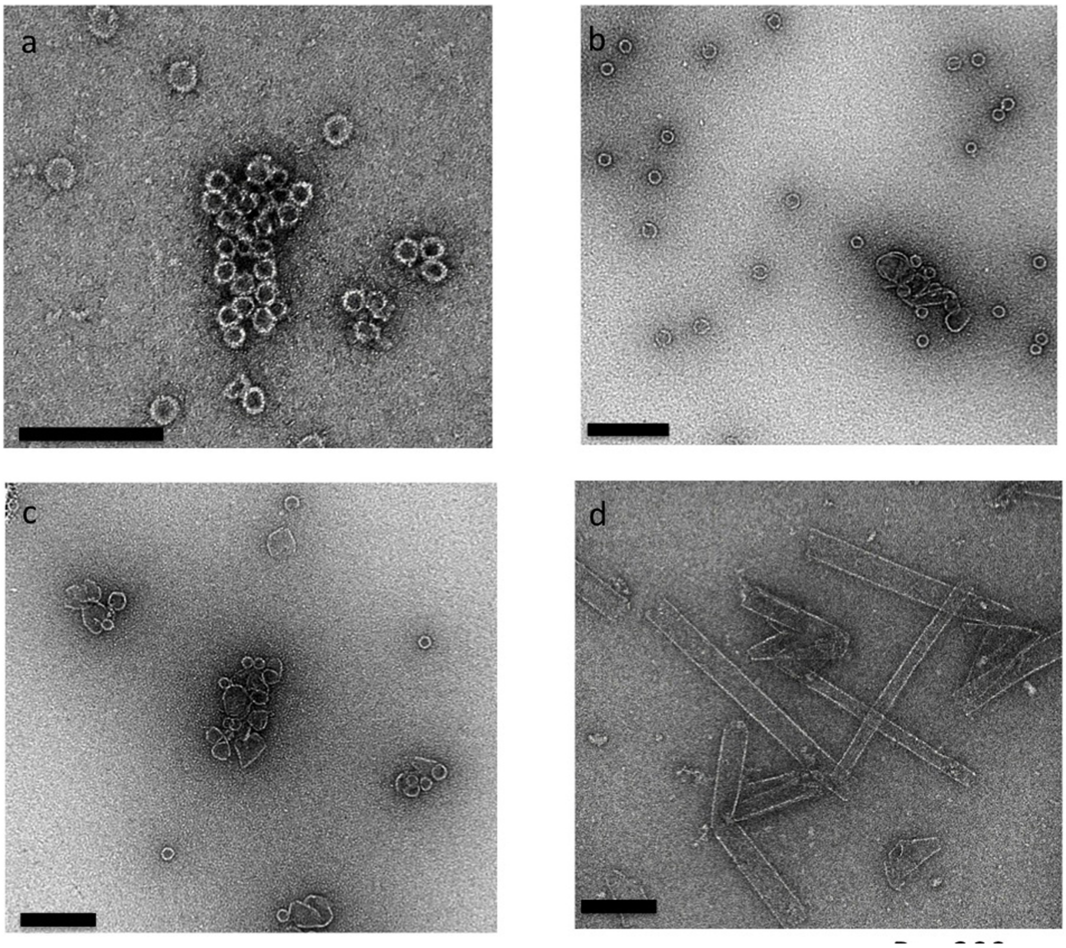

Figure S2. HBc assembly induced by BAY 41-4109 at different BAY 41-4109/HBc dimer molar ratios

(a) No drug; (**b**) 1:6; (**c**) 1:5; (**d**) 1:3. In all images, scale bars represent 200 Å.

a

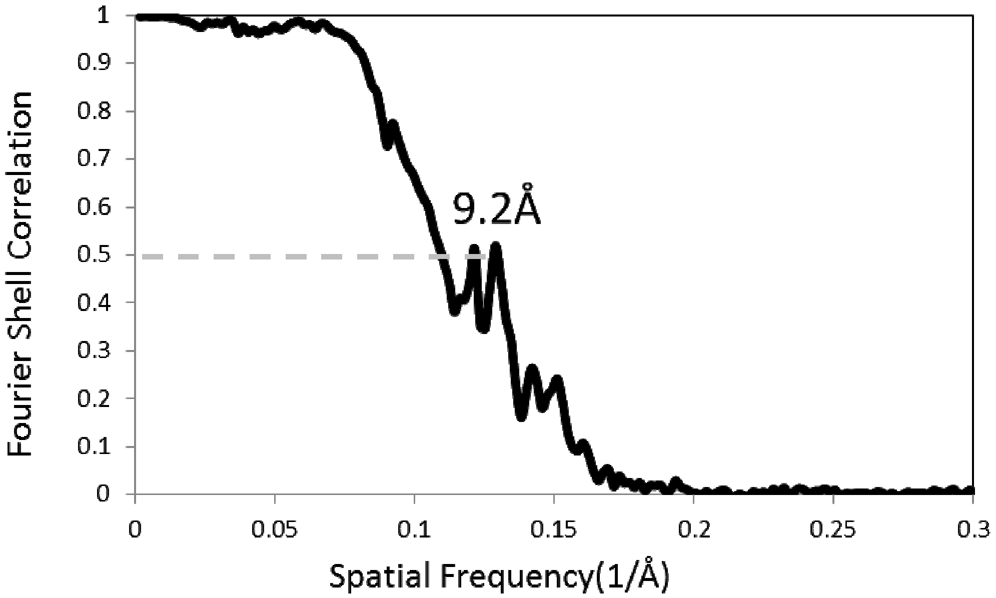

b

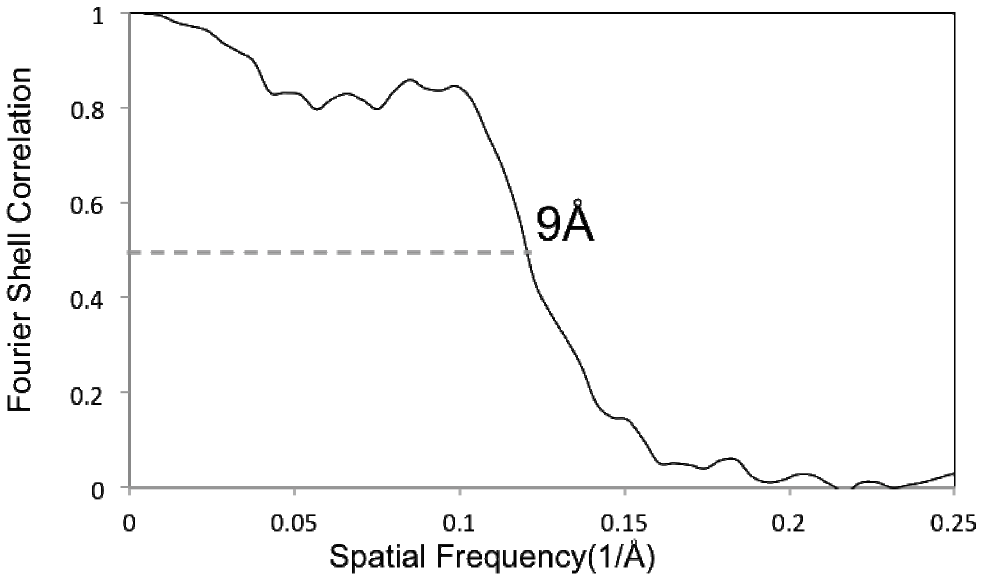

Figure S3. Resolution assessment of the reconstructed HBc tubular ensemble

(a) The resolution of the EM map is estimated as 9.2 Å according to Gold standard Fourier shell correlation (FSC) 0.5, consistent with the FSC calculated between model and map at 0.5 in (b).

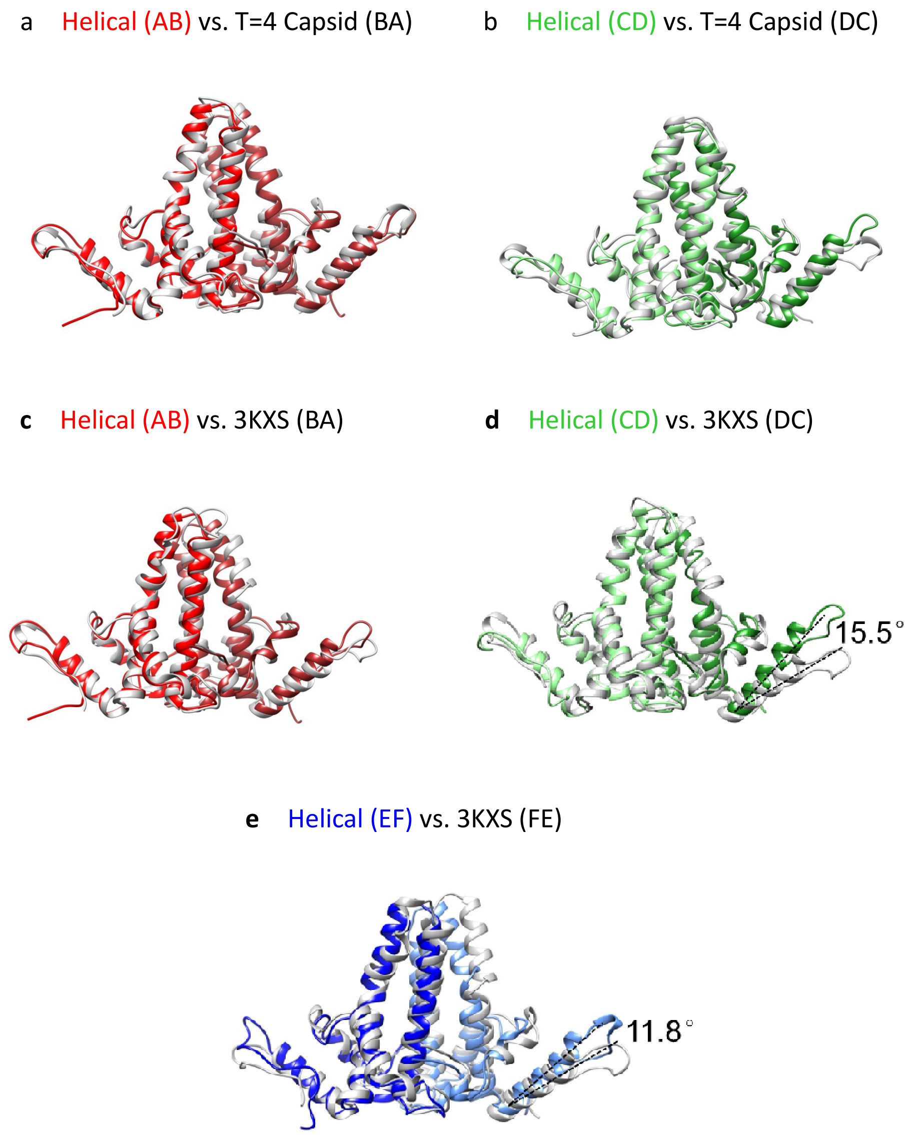

Figure S4. Structural comparisons between HBc dimers in tube, capsid and sheet-like ensemble

(a) & (**c**) Overlays of HBc AB dimer in tube with HBc BA dimer in capsid and sheet-like ensemble (3KXS).

(**b**) & (**d**) Overlays of HBc CD dimer in tube with HBc DC dimer in capsid and sheet-like ensemble (3KXS).

(**e**) Overlay of HBc EF dimer in tube with HBc FE dimer in sheet-like ensemble (3KXS).

The significant conformational differences are marked with the helix shift angles labelled. It is noteworthy that the structural variance appears different from the overlays between the same type HBc dimers in the same orientations (i.e., AB vs. AB, CD vs. CD, EF vs. EF) shown in Figure 4, indicating that the HBc dimer structure is asymmetrical.

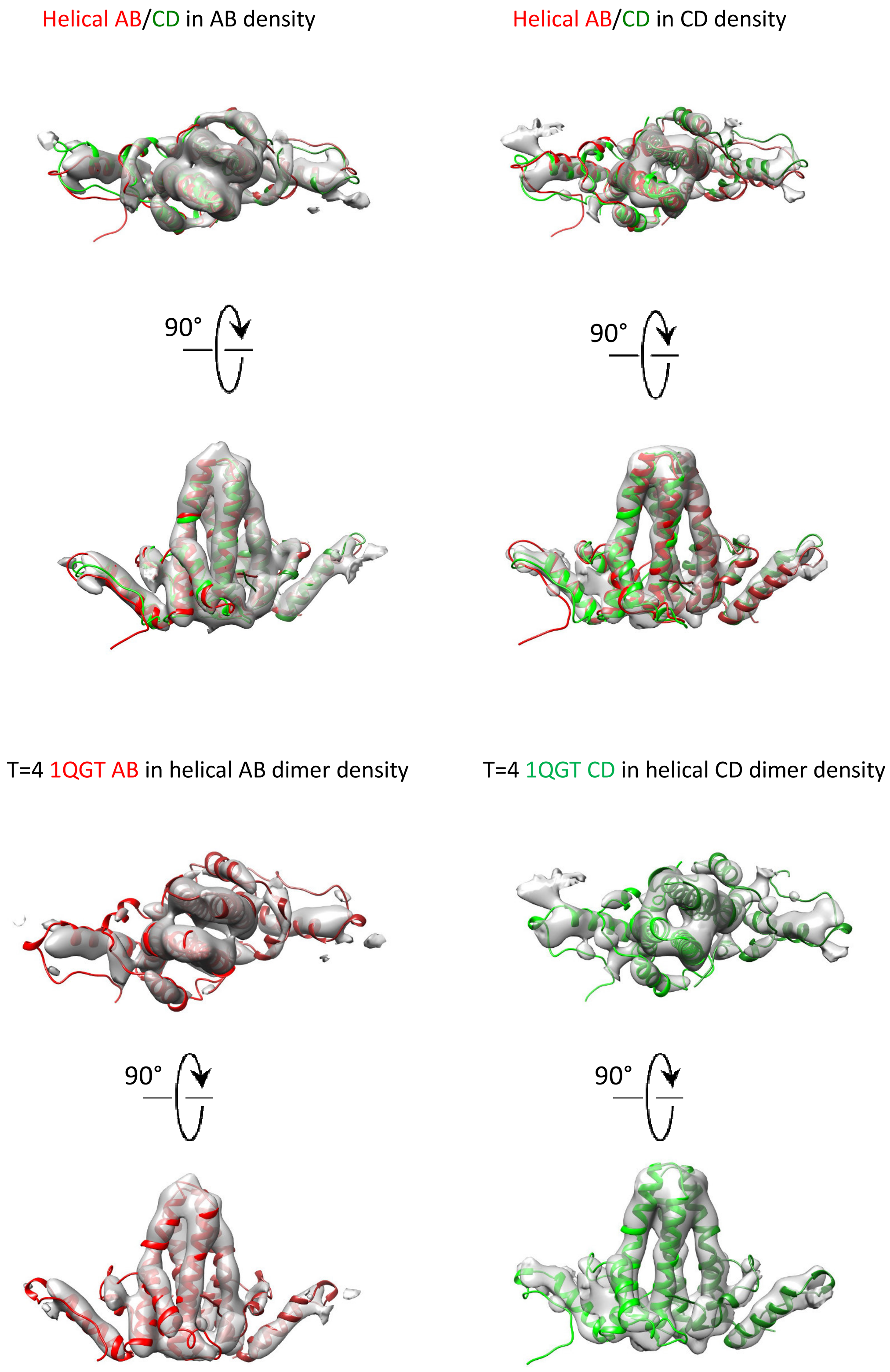

Figure S5. Structural model fitting of HBc dimers into dimer density maps of helical tube

a

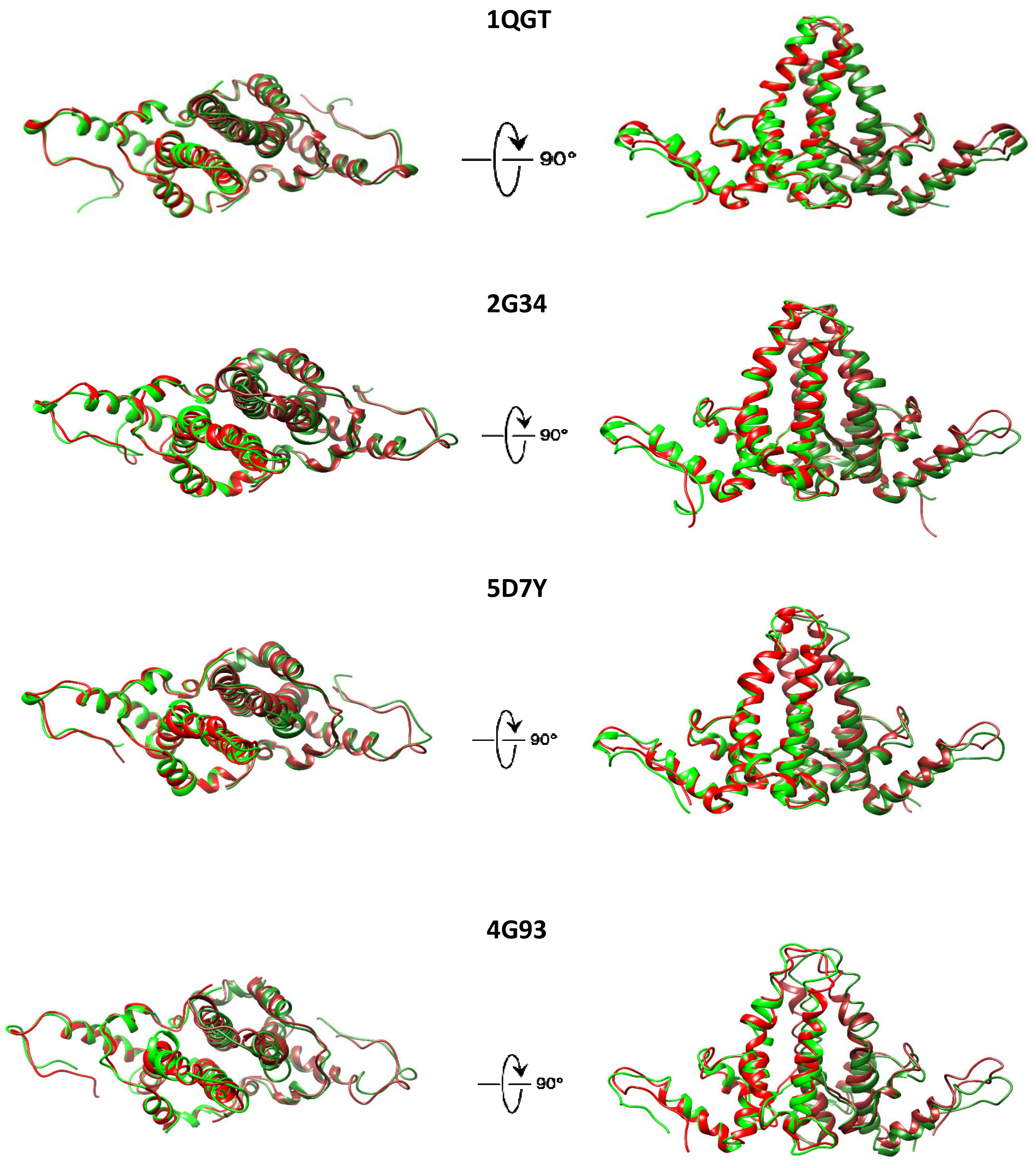

Figure S6. Structural comparisons of HBc dimers from different origin

(a) Overlays of HBc dimers (AB vs. CD) within the apo-capsid (1QGT) and drug-induced capsid (2G34 or 5D7Y or 4G93). Only subtle and minor conformational differences are observed for helix α5.

b

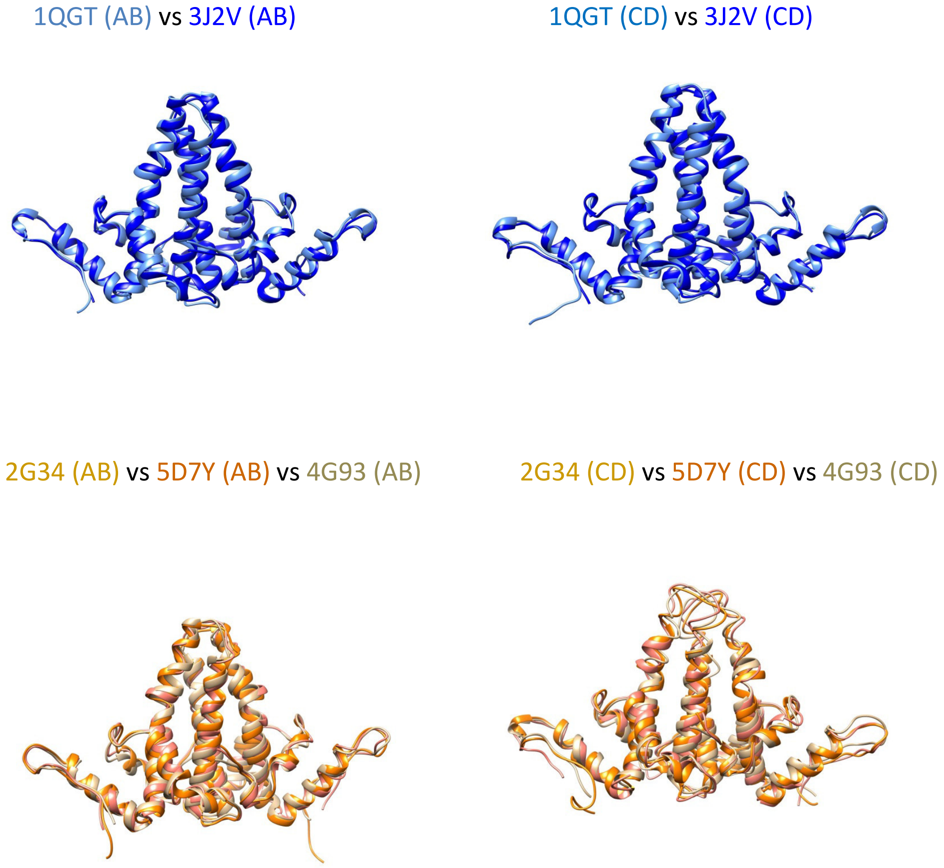

Figure S6. Structural comparisons of HBc dimers of different origin (continued)

(b) Overlays of the same type of HBc dimers between apo- (upper panel) or drug-induced capsids (lower panel). Only subtle and minor conformational differences are observed.

c

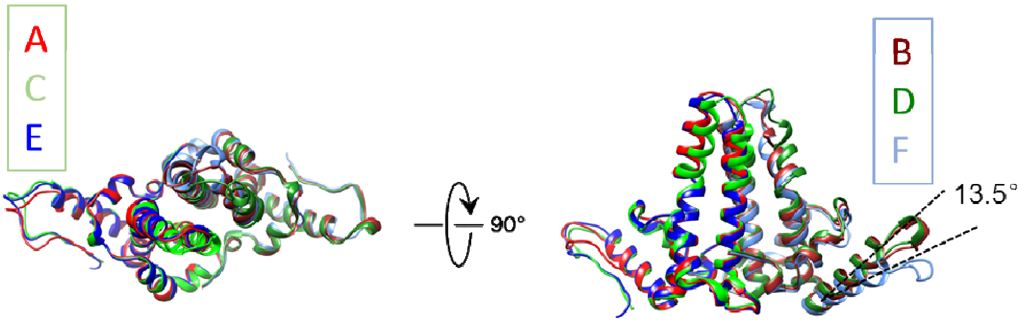

Figure S6. Structural comparisons of HBc dimers of different origin (continued)

(c) Overlays of HBc dimers (AB vs. CD vs. EF) within the sheet-like ensemble (3KXS). Subtle but major conformational differences are apparent, particularly helix α5. The significant conformational differences are marked with the helix shift angle labelled.

d

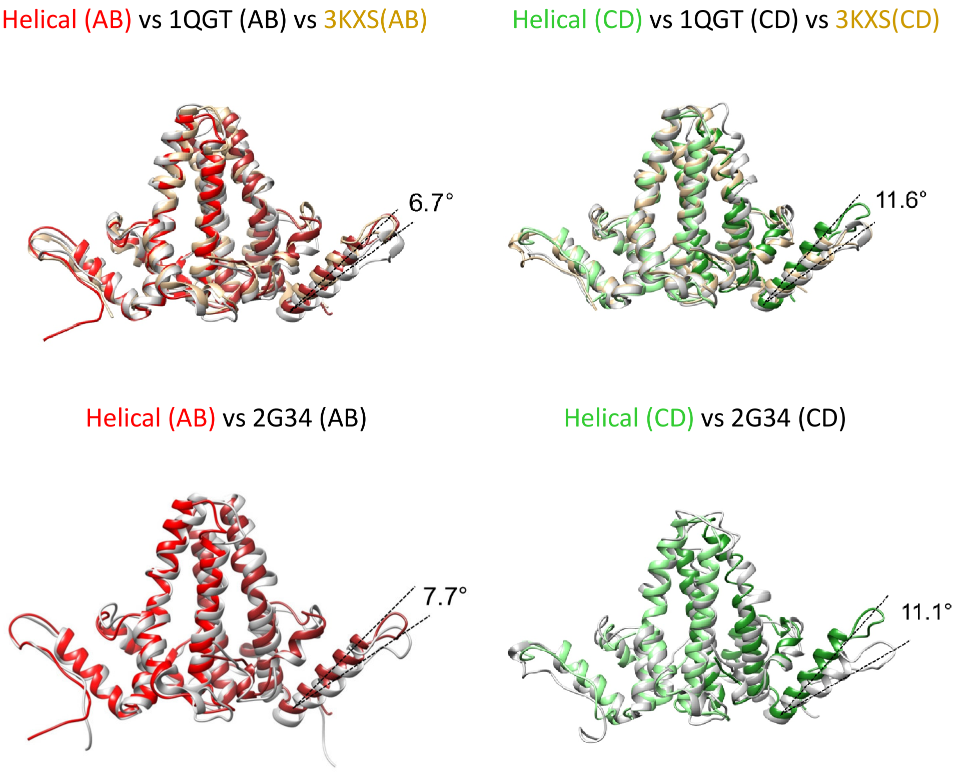

Figure S6. Structural comparisons of HBc dimers of different origin (continued)

(d) Overlays of the same type of HBc dimers from different kind of HBc ensembles (helical tube vs. capsid vs. sheet). Subtle but major conformational differences are apparent, particularly helix $\alpha5$. The significant conformational differences are marked with the helix shift angles labelled.

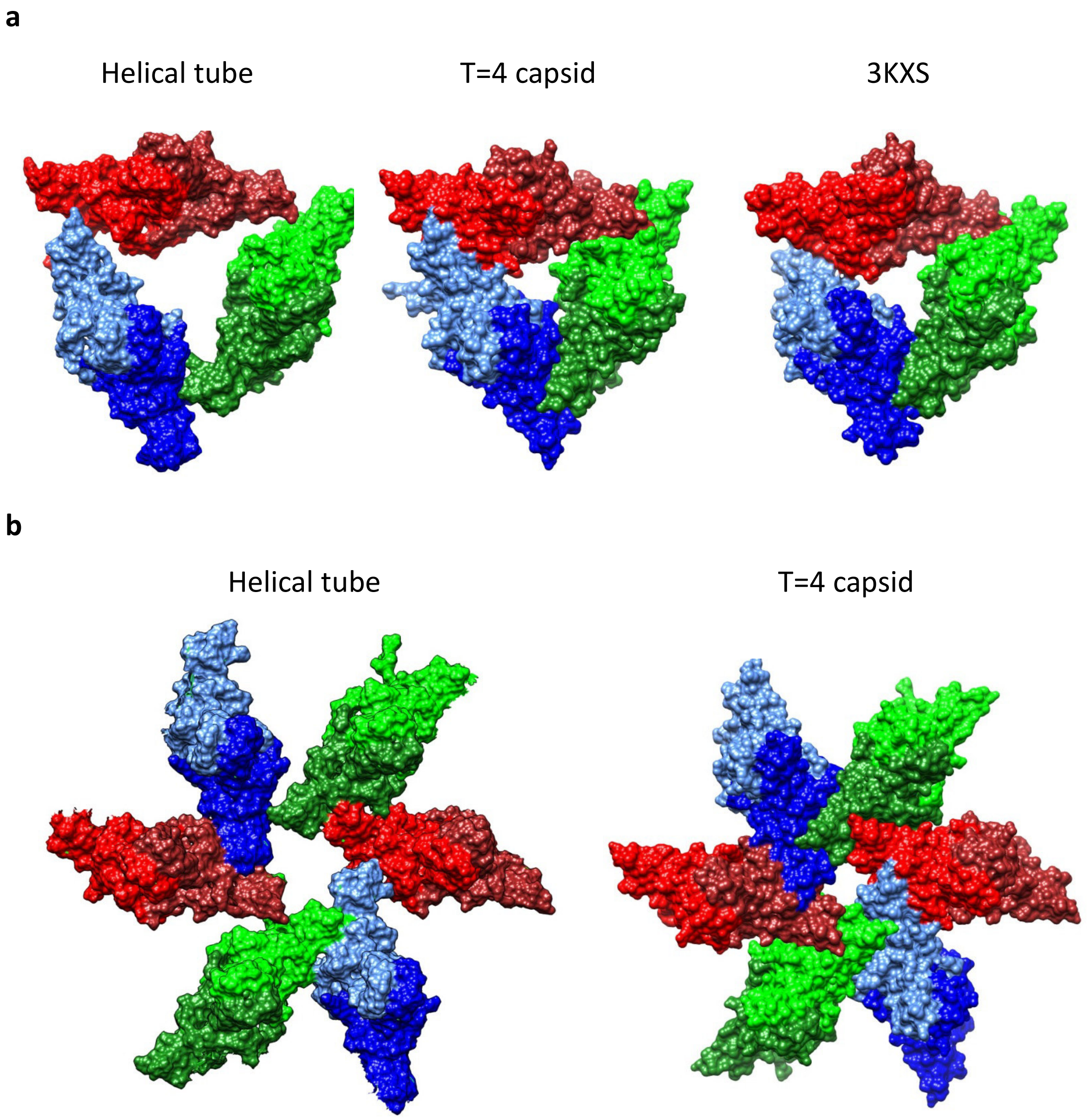

Figure S7. Interactions of HBc dimers manifested as dimer-dimer contact in tube, capsid and sheet-like ensemble at quasi 3-fold and quasi 6-fold axes

The structures are displayed as space-filled models to show the contact area between HBc dimers. The contact area between HBc dimers in helical tube is apparently smaller than in T=4 capsid and in sheet-like ensemble around both the quasi 3-fold (**a**) and quasi 6-fold (**b**) axes.

Table S1. Relative angle displacement of helix α5		
Apo-capsid		
1QGT (AB) vs 3J2V (AB)	A/A 2.8°	B/B 1.8°
1QGT (CD) vs 3J2V (CD)	C/C 1.6°	D/D 1.9°
Drug-induced capsid		
2G34(AB) vs 5D7Y(AB) vs 4G93(AB)	A/A 2.1°	B/B 2.9°
2G34(CD) vs 5D7Y(CD) vs 4G93(CD)	C/C 2.2°	D/D 3.7°
Helical tube vs T=4 capsid		
Helical tube (AB) vs 1QGT(AB)	A/A 9.8°	B/B 7.8°
Helical tube (CD) vs 1QGT(CD)	C/C 3.6°	D/D 8.2°
Sheet vs T=4 capsid		
3KXS (AB) vs 1QGT(AB)	A/A 2.7°	B/B 3.2°
3KXS (CD) vs 1QGT(CD)	C/C 11.5°	D/D 5.4°
